# Une localisation rare de la tuberculose: la tuberculose endométriale

**DOI:** 10.11604/pamj.2019.33.45.17520

**Published:** 2019-05-21

**Authors:** Anthelme Kouessi Agbodande, Roger Leoubou Dodo, Aoulath Issa, Sonia Adjadohoun, Angèle Azon-Kouanou, Armand Finangnon Wanvoegbe, Mohamed Djivèdé Akanni, Roberto Prudencio, Djimon Marcel Zannou, Fabien Houngbe

**Affiliations:** 1 Centre National Hospitalier et Universitaire Hubert Koutoukou Maga, Cotonou, Bénin; 2 Clinique de la Patte d’Oie, Cotonou, Bénin; 3 Centre Hospitalier Universitaire Départemental de Ouémé Plateau, Porto Novo, Bénin

**Keywords:** Tuberculose, endomètre, Cotonou, Tuberculosis, endometrium, Cotonou

## Abstract

La tuberculose demeure un problème de santé publique, surtout dans les pays en voie de développement. Si la forme pulmonaire bacillaire est la plus fréquente, la forme génitale est rare et sous-diagnostiquée. Nous rapportons un cas de tuberculose de l’endomètre. Il s’agit d’une patiente de 72 ans ayant une hémoglobinopathie SC qui a consulté son gynécologue pour des leucorrhées trainantes. L’examen cytobactériologique des prélèvements vaginaux a mis en évidence un *Stréptococcus agalactatiae*. Malgré une antibiothérapie adaptée, l’évolution a été marquée par une persistance des leucorrhées. L’échographie pelvienne a objectivé un endomètre épaissi avec une image hyperéchogène du fond de l’utérus, évocatrice de tumeur de l’endomètre. L’examen anatomopathologique des pièces de curetage biopsique de l’endomètre a conclu à une endométrite granulomateuse en faveur d’une tuberculose folliculaire. L’évolution a été favorable sous traitement antituberculeux. La tuberculose génitale n’est pas exceptionnelle et doit être évoquée devant une leucorrhée persistante malgré un traitement adapté en milieu d’endémie tuberculeuse.

## Abstract (en)

Tuberculosis is a public health problem, especially in the developing countries. Bacillary pulmonary tuberculosis is the most common form of tuberculosis while genital tuberculosis is rare and underdiagnosed. We here report a case of endometrial tuberculosis. The study involved a 72-year old female patient with SC hemoglobinopathy consulting her gynecologist because of chronic leukorrhea. Cytobacteriologic assessment of vaginal swabs showed Streptococcus agalactatiae. Despite suitable antibiotic therapy, the evolution was marked by the persistence of leukorrhea. Pelvic ultrasonography objectified thickened endometrium, appearing as hyperechoic at the base of the uterus. This was suggestive of endometrial cancer. Anatomo-pathological examination of endometrial curettage samples showed granulomatous endometritis suggesting follicular tuberculosis. Outcome was favorable under antituberculosis treatment. In TB endemic areas, genital tuberculosis is not exceptional and should be suspected in patients with chronic leukorrhea despite suitable treatment.

## Introduction

La tuberculose (TB) est fréquente dans le monde avec neuf millions de nouveaux cas et deux millions de décès enregistrés annuellement [1]. Elle est une des maladies infectieuses les plus répandues dans le monde et constitue un problème majeur de santé publique, surtout dans les pays à moyens limités [2-4]. Au Bénin en 2015, l’incidence de la TB était de 60/100.000 habitants [5]. Alors que la tuberculose pulmonaire, forme la plus fréquente d’atteinte bacillaire, constitue une préoccupation majeure des services de prise en charge, les formes extra-pulmonaires, particulièrement la forme génitale de cette affection demeure sous-estimée [6]. Selon Ravelosoa *et al.* [6], la tuberculose génitale est estimée à 0,2% de l’ensemble des localisations tuberculeuses. Ces formes génitales sont considérées comme formes graves parce qu’elles peuvent se compliquer d’infertilité [1]. La plus connue de ces formes génitales est l’atteinte des trompes. L’atteinte de l’endomètre reste peu décrite. Nous rapportons un cas de tuberculose endométriale qui illustre les difficultés diagnostiques des formes génitales. Ce cas sensibilise le clinicien à avoir présent à l’esprit la possibilité d’une tuberculose endométriale chez une patiente qui consulte pour des symptômes gynécologiques banals.

## Patient et observation

Une patiente de 72 ans hémoglobinopathie SC, gestité 8; parité 5 et ménopausée depuis 19 ans a consulté pour des leucorrhées abondantes persistantes. Le début remontait à deux mois avant la consultation par des leucorrhées blanchâtres, non prurigineuses, abondantes, épaisses, gluantes, non malodorantes. L’interrogatoire ne retrouve pas de notion de fièvre ou d’amaigrissement et elle ne signale ni douleurs pelviennes, ni métrorragies. Il n’a pas été retrouvé de notion de contage tuberculeux.

**Figure 1:**
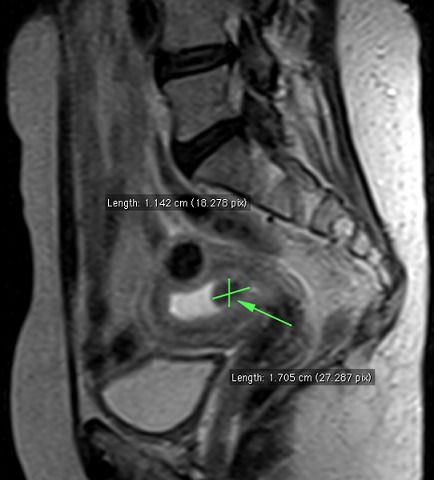
IRM (imagerie par résonance magnétique) pelvienne, séquence pondérée T2 en coupe sagittale, montrant une formation ovalaire en hyposignal dans la région isthmique de 11,4mmx17mm avec une rétention liquidienne endocavitaire au niveau de l’utérus

Devant ce tableau clinique, une infection génitale était évoquée. Un prélèvement de leucorrhée pour examen cytobactériologique et culture avec antibiogramme a été réalisé. La culture a mis en évidence un *Stréptococcus agalactatiae* sensible à l’association amoxicilline + acide clavulanique. Elle était mise sous cet antibiotique associé à un traitement locale intra-vaginale (polyginax ovule, colposeptine). L’évolution a été marquée par une persistance des leucorrhées. Une échographie pelvienne a été réalisée et a noté un endomètre épaissi avec la présence d’une image hyperéchogène de 15,1mm x 15,6 mm au niveau du fond utérin. Une imagerie par résonance magnétique (IRM) réalisée a noté la présence d’une structure tissulaire endocavitaire de l’utérus, de taille évaluée à 11,4 mm x 17 mm réalisant une rétention endocavitaire de l’utérus avec absence d’anomalies au reste du pelvis et de l’’etage abdominal (Figure 1). Une cause néoplasique a été suspectée justifiant un curetage biopsique de l’endomètre avec examen anatomo-pathologique. Cet examen retrouvait: « des fragments de muqueuses endométriale; siège d’un tissu de granulation formé de nappes de lymphocytes et de plasmocytes avec de nombreux histiocytes. On retrouvait également des cellules en amas parfois folliculaires, de cellules épithélioides englobant quelques cellules géantes multi nucléées de type Langhans. Une présence de matériel à éosinophile d’allure caséeux ».

Une endométrite granulomateuse d’origine tuberculeuse a été retenue. La recherche des bacilles acido-alcoolo-résistants dans les leucorrhées était négative et la radiographie pulmonaire était normale. Sous traitement anti tuberculeux (2 mois d’une association faite d’éthambutol, rifampicine, isoniazide et pyrazinamide et 4 mois de rifampicine associée à l’isoniazide), l’évolution a été favorable avec une disparition totale des leucorrhées et de la masse.

## Discussion

L'incidence de la tuberculose génitale n’est pas connue étant donné que beaucoup de cas restent non diagnostiqués à cause de la fréquence des formes latentes et inapparentes [7]. Elle est toujours secondaire et succède soit à une dissémination par voie hématogène à partir d’un foyer tuberculeux initial, avec une atteinte initiale des trompes (100% des cas) réalisant un tableau de salpingite à partir de laquelle l’infection progresse vers les autres organes génitaux; soit à une contamination par voie lymphatique à partir des ganglions pelviens; de rares cas secondaires à une inoculation directe par contage vénérien ont été rapportés par Weinstein [8-10]. Elle touche de façon prédominante la femme jeune, en pleine activité génitale [8, 11]. Il existe des formes déclarées en péri- ou post-ménopausique. Notre patiente était âgée de 72 ans au moment du diagnostic. Chau [12] et Houda [7] ont publié également des cas similaires. Cette endométrite tuberculeuse de la femme ménopausée est souvent isolée sans atteinte tubaire contrairement à celui de la femme jeune qui est souvent associée à une salpingite tuberculeuse [12]. Les circonstances de découverte de la tuberculose génitale féminine sont très variées. Les motifs de consultation les plus rapportées étaient l´infertilité (44%), la douleur pelvienne (25%), le saignement vaginal (18%), l´aménorrhée (5%), la leucorrhée (4%) et les métrorragies post-ménopausiques (2%) [13]. Le mode révélateur de notre patiente était les leucorrhées persistantes.

Il s’avère donc important d’avoir présent à l’esprit la possibilité de la tuberculose endométriale chez une patiente qui présente des leucorrhées trainantes ne cédant pas avec un traitement antibiotique bien conduit en zone endémique de tuberculose. Le diagnostic n’est pas évident, faisant appel à des techniques d’imagerie, des examens biologiques et histologiques. A l’imagerie, la radiographie pulmonaire peut mettre en évidence des séquelles parenchymateuses ou pleurales [7, 11]. L’hystérosalpingographie, examen de choix montre souvent deux aspects, caractéristiques de l’origine tuberculeuse : les synéchies utérines et l’image de passage vasculaire qui donne le classique angiogramme de KIKA [8]. Chez la patiente, compte tenu de l’âge et du tableau clinique, le premier diagnostic évoqué était une cause néoplasique. Cela explique pourquoi cet examen n’était pas fait chez la patiente. Mais il faut souligner que des associations de tumeur et de tuberculose endométriale ont été décrites [14]. Le diagnostic de certitude peut être direct par la mise en évidence du mycobacterium tuberculosis à l’examen direct microscopique, soit après mise en culture de prélèvements pathologiques. Il peut être également indirect par analyse anatomopathologique du matériel obtenu par curetage biopsique endométrial [6, 10, 11]. Dans notre cas le diagnostic était indirect.

Le traitement est actuellement bien codifié et repose sur l’administration quotidienne pendant 6 mois d’isoniazide et de rifampicine, associée pendant les 2 premiers mois de pyrazinamide et éthambutol [6]. La surveillance clinique et paraclinique s’effectue régulièrement tout au long du traitement. Le traitement chirurgical de la forme génitale est indiquée en cas de: persistance de masses annexielles malgré le traitement médical, en particulier en cas d´abcès froid, de rechute de la tuberculose endométriale après une année de traitement, de persistance des douleurs pelviennes après 3 mois de traitement ou lorsqu’elles n’ont pas totalement disparu au terme d´un an de traitement, en cas de métrorragies persistant après guérison anatomique et clinique, de fistules qui ne se tarissent pas [6]. Notre patiente sous traitement antituberculeux avait une bonne évolution avec un tarissement des leucorrhées. Il n’y avait pas d’indication à un traitement chirurgical.

## Conclusion

La tuberculose demeure fréquente mais se manifeste rarement par une atteinte endométriale. Les femmes jeunes de bas niveau socioéconomique, consultant devant une stérilité sont le plus concernées. Il faut cependant savoir l’évoquer devant une symptomatologie pelvienne traînante, quel que soit l’âge, et faire des examens concourant au diagnostic.

## Conflits d’intérêts

Les auteurs ne déclarent aucun conflit d’intérêts.
